# A framework to assess the impact of number of trials on the amplitude of motor evoked potentials

**DOI:** 10.1038/s41598-020-77383-6

**Published:** 2020-12-08

**Authors:** Claudia Ammann, Pasqualina Guida, Jaime Caballero-Insaurriaga, José A. Pineda-Pardo, Antonio Oliviero, Guglielmo Foffani

**Affiliations:** 1 HM CINAC, Hospital Universitario HM Puerta del Sur, HM Hospitales, Universidad CEU-San Pablo, Madrid, Spain; 2 CIBERNED, Instituto de Salud Carlos III, Madrid, Spain; 3 Hospital Nacional de Parapléjicos, Toledo, Spain

**Keywords:** Transcranial magnetic stimulation, Biostatistics

## Abstract

The amplitude of motor evoked potentials (MEPs) elicited by transcranial magnetic stimulation (TMS) is a common yet highly variable measure of corticospinal excitability. The tradeoff between maximizing the number of trials and minimizing experimental time remains a hurdle. It is therefore important to establish how many trials should be used. The aim of this study is not to provide rule-of-thumb answers that may be valid only in specific experimental conditions, but to offer a more general framework to inform the decision about how many trials to use under different experimental conditions. Specifically, we present a set of equations that show how the number of trials affects single-subject MEP amplitude, population MEP amplitude, hypothesis testing and test–retest reliability, depending on the variability within and between subjects. The equations are derived analytically, validated with Monte Carlo simulations, and representatively applied to experimental data. Our findings show that the minimum number of trials for estimating single-subject MEP amplitude largely depends on the experimental conditions and on the error considered acceptable by the experimenter. Conversely, estimating population MEP amplitude and hypothesis testing are markedly more dependent on the number of subjects than on the number of trials. These tools and results help to clarify the impact of the number of trials in the design and reproducibility of past and future experiments.

## Introduction

Transcranial magnetic stimulation (TMS) is a safe, non-invasive technique based on delivering electromagnetic pulses to the cerebral cortex through a magnetic coil inducing a focused electric field in the underlying brain tissue^1^. When a single pulse of TMS is applied to the primary motor cortex with sufficient intensity, it depolarizes corticospinal neurons, eliciting a muscle contraction in the contralateral peripheral muscles, known as motor evoked potential (MEP)^1–3^. The peak-to-peak amplitude of MEPs recorded by surface electromyography (EMG) is commonly used to quantify the level of corticospinal excitability^4,5^. In the last few decades, TMS-induced MEPs have been increasingly used to obtain neurophysiological information about human motor function and mechanistic insights into neurological disorders^6–8^.

Unfortunately, MEP amplitude displays high trial-to-trial variability, owing to both experimental and biological factors^9^. The intrinsic fluctuations in MEP amplitude depend on the state of ongoing oscillatory activity of cortical neurons beneath the TMS coil^10–12^ and on the changing synchronization of motor neuron discharges at the spinal level^13^. Experimental factors like location on the scalp^14^, coil orientation^15^, stimulus intensity^9,16^, and probably small changes in coil positioning (tilt, roll and twist) are also linked to MEP amplitude variability. The level of attention^17^, and muscle activation of the subject^16,18^ additionally affect MEP amplitude and variability. In population terms, MEP variability also depends on gender and age^19^. Even though some of these factors may be partly controlled by careful experimental designs and, to some extent, by the use of neuronavigation^20,21^, MEP amplitude remains a substantially variable measure.

A common strategy to deal with trial-to-trial variability is to model MEP amplitude as a stochastic variable whose “true” probability distribution depends on all the possible sources of experimental and biological variability. Consequently, even though the “true” MEP amplitude does not exist in reality, its expected value can be estimated by averaging over trials. When planning a TMS experiment, therefore, a basic methodological question always arises: how many trials should be used? One might simply say: the more, the better. However, experimental time is often limited and the biological and experimental conditions—and thus the “true” probability distribution—are likely to change over time. For example, an experiment might be designed to capture a biological phenomenon that is a priori delimited in time. Likewise, it may be difficult to guarantee stable attention and arousal during long experiments, and long protocols may even induce complex metaplasticity/anti-gating effects^22,23^. Estimating MEP amplitude thus becomes a tradeoff between maximizing the number of trials and minimizing the experimental time. Could 10 trials be enough? Or 20, or 30? What would be gained by using 100? In other words, what is the estimation error expected with a given number of trials? Should the same number of trials be used in a single-case study and in an experiment with 100 subjects? And does the number of trials have the same impact on statistical comparisons with independent-measures vs. repeated-measures designs? Recent studies have been designed to provide rule-of-thumb empirical answers to some of these questions, specifically to estimate single-subject MEP amplitude in certain experimental conditions^24–31^. However, since MEP variability depends on many experimental and biological sources and of the specific TMS technique employed, rule-of-thumb answers (e.g. at least 30 trials) are unlikely to fit all experimental situations. Here we aim to offer a more general theoretical framework to inform the decision about how many trials to use in TMS experiments.

The manuscript is organized as follows. First, we provide an analytical demonstration that some empirical approaches used in previous studies to define the minimum number of trials to estimate MEP amplitude^26–29,31^ have limited implications. We then present a more principled general framework—derived from basic statistical reasoning—that clarifies the impact of number of trials on single-subject MEP amplitude, population MEP amplitude, hypothesis testing and test–retest reliability. We subsequently validate the equations with Monte Carlo simulations. Next, we apply the proposed framework in two experimental datasets. We first recorded 100 MEP trials in 20 subjects to provide a step-by-step application of the equations to estimate single-subject MEP amplitude and population MEP amplitude (Experiment 1). We then use the data from Experiment 1 to define the optimal number of trials and subjects to be used in a representative experiment designed to detect significant MEP amplitude differences between two stimulus intensities commonly employed in stimulus–response curves^9,16,26^ (110% of the resting motor threshold [RMT] vs. 120%RMT) (Experiment 2). Beyond the specific examples, the equations and reasoning have general validity, so they can be used in a variety of experimental designs.

## Results

### Analytical results

#### Number of trials for estimating single-subject MEP amplitude: previous studies

In a hypothetical single-pulse TMS experiment in which MEP amplitude is collected for ${n}_{max}$ trials in single subjects, the cumulative average ${\widehat{\mu }}_{trials}(n)$ is defined as the average MEP amplitude obtained with the first *n* trials, so that ${\widehat{\mu }}_{trials}\left({n}_{max}\right)$ is the sample average with all trials. For simplicity, we will refer to the sample average (with ${n}_{max}$ trials) simply as ${\widehat{\mu }}_{trials}$. Previous studies empirically defined the optimal number of trials for estimating single-subject MEP amplitude as the minimum number of trials ${n}_{opt}$ that allows the cumulative average to come within a certain level of ‘acceptable similarity’ to the sample average. Two main measures of ‘acceptable similarity’ were used: (i) a 95% confidence interval (${n}_{opt\_ci}$)^26–29,31^, and (ii) a ± 10% difference (${n}_{opt\_\%diff}$)^28^ around the sample average.

We can define the inclusion of the cumulative average within the desired level of acceptable similarity as a probability of inclusion ${p}_{incl}$, so that $\alpha =1-{p}_{incl}.$ With central limit theorem assumptions, here we show that both ${n}_{opt\_ci}$ and ${n}_{opt\_\%diff}$ are analytical functions of ${n}_{max}$, namely:

1 $${n}_{opt\_ci}=\frac{{n}_{max}}{1+{\left(\frac{{z}_{1-{\alpha }_{ci}/2}}{{z}_{1-{\alpha }/2}}\right)}^{2}},$$

2 $${n}_{opt\_\%diff}=\frac{1}{\frac{1}{{n}_{max}}+{\left(\frac{{\eta \widehat{\mu }}_{trials}}{{z}_{1-{\alpha }/2} {\widehat{\sigma }}_{trials}}\right)}^{2}},$$

where ${z}_{1-{\alpha }_{ci}/2}$ is the critical value of the standard normal distribution for a confidence interval of $1-{\alpha }_{ci}$ (e.g. for a 95% c.i., ${\alpha }$ = 0.05 and ${\text{z}}_{1-{\alpha }/2 }$ = 1.96), ${z}_{1-{\alpha }/2}$ is the critical value corresponding to the probability of inclusion ${p}_{incl},$ $\eta$ is the relative error that defines the acceptable difference from the sample average (e.g. for ± 10%, $\eta$ = 0.1), ${\widehat{\mu }}_{trials}$ and ${\widehat{\sigma }}_{trials}$ are the sample average and standard deviation across trials (computed with ${n}_{max}$). For derivations, see “Methods”. Unfortunately, ${n}_{opt\_ci}$ does not depend on the variability ${\widehat{\sigma }}_{trials}$, and for both ${n}_{opt\_ci}$ and ${n}_{opt\_\%diff}$ the cumulative average is a priori bound to reach the required ‘acceptable similarity’ to the sample average with a number of trials that depends on and is upper bounded by the total number of trials available ${n}_{max}$.

In Eq. (1), the definition of the optimal number of trials ${n}_{opt\_ci}$ is solely a function of ${n}_{max}$*,* ${z}_{1-{\alpha }_{ci}/2}$ and ${z}_{1-{\alpha }/2}$*.* The above-cited studies were empirically trying to define the minimum number of trials ${n}_{opt\_ci}$ that allowed the cumulative average to come within a 95% confidence interval around the sample average. They thus assumed ${\alpha }_{ci}$ = 0.05, which implies ${z}_{1-{\alpha }_{ci}/2}$ = 1.96. They were also using ${p}_{incl}=1$, which would correspond to a theoretical ${z}_{1-{\alpha }/2}$ =  + ∞, but in practice corresponded to an arbitrary ${p}_{incl}$ < 1 due to the finite number of subjects. For example, if ${z}_{1-{\alpha }/2}$ = 2.493, which corresponds to an arbitrary but mathematically elegant inclusion probability ${p}_{incl}$ between 0.95 and 0.99, then ${n}_{opt\_ci}={n}_{max}$/φ, where φ = 1.618 is the golden ratio. With this ‘golden’ inclusion probability, the ‘optimal’ number of trials estimated by the inclusion of the cumulative average within a 95% confidence interval around the true average would be ${n}_{opt\_ci}$ = 19, 25 and 62 with ${n}_{max}$ = 30, 40 and 100, respectively. With an empirical ${p}_{incl}=1$, as used in previous studies^26–29,31^, if the number of subjects increases, then the experimental estimate of ${n}_{opt\_ci}$ asymptotically tends to ${n}_{max}$.

Unlike Eq. (1), Eq. (2) does take into account the trial-to-trial variability of MEP amplitude ${\widehat{\sigma }}_{trials}$. Unfortunately, however, it still depends on (and is limited by) the total number of trials available ${n}_{max}$. For example, if ${\widehat{\mu }}_{trials}$ = 1, ${\widehat{\sigma }}_{trials}$ = 0.5, $\eta$ = 0.1 and ${z}_{1-{\alpha }/2}$ = 2.493, then ${n}_{opt\_\%diff}$ = 25, 32 and 61 with ${n}_{max}$ = 30, 40 and 100, respectively. With ${p}_{incl}=1$ as previously used empirically^28^, if the number of subjects increases, then the experimental estimate of ${n}_{opt\_\%diff}$ also asymptotically tends to ${n}_{max}$.

#### Number of trials for estimating single-subject MEP amplitude: a principled framework

In order to avoid the limitations of previous empirical studies attempting to define the number of trials for estimating single-subject MEP amplitude, we rescue a more principled measure of ‘acceptable similarity’ that had already been used in the early TMS literature^14^: the inclusion of the cumulative average within an acceptable difference (e.g. ± 10%) from the *true* average. The optimal number of trials ${n}_{opt}$ for estimating single-subject MEP amplitude is thus simply obtained as the number of trials at which the confidence interval of the estimate of the true average equals the acceptable difference from the true average, i.e.

3 $${n}_{opt}={\left[\frac{{z}_{1-\alpha /2}{\sigma }_{trials}}{\eta {\mu }_{trials}}\right]}^{2}={\left[\frac{{z}_{1-\alpha /2}}{\eta }C{V}_{trials}\right]}^{2},$$

where the critical value ${z}_{1-\alpha /2}$ is now defined by the desired probability of inclusion ${p}_{incl}$ within the relative error $\eta$ around the true average ${\mu }_{trials}$, and ${CV}_{trials}$ is the corresponding coefficient of variation (i.e. ${CV}_{trials}={\sigma }_{trials}/{\mu }_{trials}$). For example, if ${CV}_{trials}$ = 0.5, then 96 trials are necessary to ensure that the estimated single-subject MEP amplitude stays within 10% of the true value ($\eta$ = 0.1) with 95% probability (${p}_{incl}$ = 0.95, ${z}_{1-\alpha /2}$ = 1.96). Crucially, in Eq. (3) ${n}_{opt}$ is not upper-bounded by the total number of trials available ${n}_{max}$. Therefore, ${n}_{opt}$ can also be rigorously estimated from experimental data (without dependence on ${n}_{max}$), by substituting the true ${CV}_{trials}$ with the sample estimate ${\widehat{CV}}_{trials}$.

Note that Eq. (3) can also be derived as the theoretical asymptotic limit of Eq. (2) for a very-large number of trials, when the sample average ${\widehat{\mu }}_{trials}$ converges to the true average ${\mu }_{trials}$:

4 $$\underset{{n}_{\mathit{max}}\to \infty }{\text{lim}}\left(\frac{1}{\frac{1}{{n}_{\mathit{max}}}+{\left(\frac{{\eta \widehat{\mu }}_{\mathit{trials}}}{{z}_{1-{\alpha }/2} {\widehat{\sigma }}_{\mathit{trials}}}\right)}^{2}}\right)={\left[\frac{{z}_{1-{\alpha }/2}}{\eta }{CV}_{trials}\right]}^{2}.$$

Equation (3) can be solved for $\eta$ to calculate the relative error (i.e. the acceptable difference from the true average) that is implicitly assumed when the MEP amplitude is estimated with a given number of trials *n*, i.e.

5 $$\eta (n)=\frac{{z}_{1-\alpha /2}}{ \sqrt{n}}{CV}_{trials}=\frac{{z}_{1-\alpha /2}}{ {\mu }_{trials}}{SE}_{trials}(n),$$

where ${SE}_{trials}\left(n\right)$ is simply the standard error of ${\widehat{\mu }}_{trials}$ estimating ${\mu }_{trials}$ with $n$ trials. The statistical error thus decreases with the inverse of the square root of $n$. For example, if ${CV}_{trials}$ = 0.5 and ${z}_{1-\alpha /2}$ = 1.96, then reducing the number of trials $n$ from 96 to 30 or 20 increases the relative error $\eta$ from 10.0% to 17.9% and 21.9%, respectively.

#### Number of trials for estimating population MEP amplitude

In many studies the objective may be to estimate the average MEP amplitude of a population of $N$ subjects, which we will refer to as the population MEP amplitude.

Substituting trials with subjects, Eq. (5) remains valid to calculate the relative error $\eta (N)$ that is assumed acceptable when the population MEP amplitude ${\mu }_{subjects}$ is estimated with *N* subjects, given the coefficient of variation across subjects ${CV}_{subjects}$ or the standard error of the population MEP amplitude ${SE}_{subjects}(N)$, i.e.

6 $$\eta \left(N\right)=\frac{{z}_{1-\alpha /2}}{ \sqrt{N}}{CV}_{subjects}=\frac{{z}_{1-\alpha /2}}{ {\mu }_{subjects}}{SE}_{subjects}(N).$$

In Eq. (6) the statistical error decreases with the inverse of the square root of the number of subjects $N$. In order to understand how the error depends on the number of trials $n$, we can decompose the variance between subjects with $n$ trials, ${\sigma }_{subjects}^{2}\left(n\right),$ into the sum of the asymptotic variance between subjects with infinite trials ${\sigma }_{subjects}^{2}$ and the error variance of the sample average within subjects due to the finite number of trials $n$^32^:

7 $${\sigma }_{subjects}^{2}(n)= {\sigma }_{subjects}^{2}+\frac{{\sigma }_{trials}^{2}}{n},$$

where ${\sigma }_{trials}^{2}$ is the MEP variance across trials, either assumed to be equal across subjects or pooled across subjects. The standard error of the population MEP amplitude then becomes

8 $${SE}_{subjects}\left(N,n\right)=\sqrt{\frac{{\sigma }_{subjects}^{2}+\frac{{\sigma }_{trials}^{2}}{n}}{N}}.$$

The relative error $\eta$ of the population MEP amplitude thus depends on the number of trials $n$ as follows:

9 $$\eta (N,n) =\frac{{z}_{1-\alpha /2}\sqrt{{\sigma }_{subjects}^{2}+\frac{{\sigma }_{trials}^{2}}{n}}}{{\mu }_{subjects} \sqrt{N}}.$$

Equations (8) and (9) show that the statistical error can be reduced by increasing either the number of trials $n$ or the number of subjects $N$. However, increasing the number of trials $n$ provides only limited benefit. For example, consider a hypothetical population of $N$ = 20 subjects with ${\mu }_{subjects}$ = 1.0 mV, ${\sigma }_{subjects}$ = 0.5 mV and ${\sigma }_{trials}$ = 0.5 mV. The minimum relative error $\eta$ achievable for estimating the population MEP amplitude with an infinite number of trials is 21.9%. If we reduce the number of trials from infinite to 10 or even 5, then the error only increases to 23.0% and 24.0%, respectively. With 10 trials, if we double ${\sigma }_{trials}$ from 0.5 to 1.0 mV, the error only increases from 23.0 to 25.9%. Conversely, the error can always be decreased by increasing the number of subjects *N*.

#### Number of trials for hypothesis testing

In many experimental situations, one might be interested in knowing if a certain number of trials is sufficient to perform hypothesis testing, for example to test if MEP amplitude is significantly different in a population of patients compared to a population of controls (unpaired), or if it is significantly different before and after an intervention on the same population of subjects (paired). The same reasoning used to estimate the population MEP amplitude can be applied to express the *t* statistic for a Student’s paired t-test as a function of the number of subjects $N$ and trials $n$:

10 $$t(N,n)=\frac{{\widehat{\mu }}_{subjects1}-{\widehat{\mu }}_{subjects2}}{\sqrt{\frac{2\left[{ \sigma }_{subjects}^{2}\left(1-r\right)+\frac{{\sigma }_{trials}^{2}}{n}\right]}{N}}},$$

where ${\widehat{\mu }}_{subjects1}$ and ${\widehat{\mu }}_{subjects2}$ are the population MEP amplitudes of the two populations to be compared, assuming for simplicity equal variances, and $r$ is the asymptotic correlation of MEPs between the two populations (i.e. the correlation that would be obtained within an infinite number of trials). Note that if we assume $r$ = 0, then Eq. (10) represents an unpaired t-test with equal $N$ and equal variances. A derivation of Eq. (10) is provided in the Methods.

The relationship between the number of trials and statistical power may be seen more directly in the corresponding formula for the calculation of the sample size ${N}_{opt}$ in a power analysis for the t-test^32^:

11 $${N}_{opt}\left(n\right)=2\frac{\left[{ \sigma }_{subjects}^{2}\left(1-r\right)+\frac{{\sigma }_{trials}^{2}}{n}\right]{\left({z}_{1-\alpha/2}+ {z}_{1-\beta}\right)}^{2}}{{\left({\mu }_{subjects1}- {\mu }_{subjects2}\right)}^{2}},$$

where $\alpha$ is the probability of type 1 error and $\beta$ is the probability of type 2 error ($1-\beta$ is the power). With typical values of $\alpha$ = 0.05 and $\beta$ = 0.20 (i.e. ${z}_{1-\alpha/2}+ {z}_{1-\beta}$ = 2.80), Eq. (11) becomes:

12 $${N}_{opt}(n)= 15.68\frac{{ \sigma }_{subjects}^{2}\left(1-r\right)+\frac{{\sigma }_{trials}^{2}}{n}}{{\left({\mu }_{subjects1}- {\mu }_{subjects2}\right)}^{2}}.$$

For example, if ${\sigma }_{subjects}$ = 0.5 mV and ${\sigma }_{trials}$ = 0.5 mV and we want to detect a difference ${\mu }_{subjects1}- {\mu }_{subjects2}$ = 0.2 mV, Eq. (12) indicates the following. In a between-subjects design ($r$= 0), with only one trial ($n$ = 1) we would need two groups of at least 196 subjects. By increasing the number of trials to $n$ = 5 or 10, the number of subjects would conveniently decrease to 118 and 108, respectively. However, further increasing the number of trials would lead to negligible additional reduction of the number of subjects needed (e.g. 103 subjects with $n$ = 20 trials, 101 subjects with $n$ = 40 trials, 98 subjects with $n$ = ∞ trials). In a within-subjects design with high correlation ($r$= 0.9), with only one trial ($n$ = 1) we would need at least 108 subjects. By increasing the number of trials to $n$ = 5, 10 or 20, the number of subjects would decrease considerably to 30, 20 and 15, respectively. Further increasing the number of trials would lead to a progressively smaller reduction of the number of subjects needed (e.g. 14 subjects with $n$ = 30 trials, 13 subjects with $n$ = 40, 10 subjects with $n$ = ∞ trials).

#### Number of trials for test–retest reliability

Finally, the number of trials $n$ clearly has an impact on the test–retest reliability of TMS measures^33^, as reported in previous experimental studies^28,31^. In the case of MEP amplitude, we can show this impact analytically. For simplicity, we focus on Pearson’s correlation, which is useful to assess test–retest reliability when only two time points are available, particularly if means and variances do not change across time points^34^. Substituting Eq. (7) in Eq. (40) (see “Methods”), the dependence of the Pearson’s correlation coefficient $r\left(n\right)$ between repeated measures on the number of trials $n$ within measures can be expressed as follows:

13 $$r\left(n\right)=r\frac{{\sigma }_{subjects}^{2}}{{\sigma }_{subjects}^{2}+\frac{{\sigma }_{trials}^{2}}{n}},$$

where $r$ and ${\sigma }_{subjects}^{2}$ are the asymptotic Pearson’s correlation across repeated measures and the (pooled) variance across subjects with infinite trials, and ${\sigma }_{trials}^{2}$ is the (pooled) variance across trials. Note that if mean and variance do not change across time points (which should be the case in the context of test–retest reliability of TMS measures), then the Pearson’s correlation coefficient is identical to the concordance correlation coefficient^34^, which in turn is virtually identical to a group of intraclass correlation coefficients that estimate the degree of absolute agreement between non-interchangeable measurements^35–37^. Equation (13) clarifies that increasing the number of trials can only increase the test–retest reliability up to a limit (i.e. $r$), which is consistent with previous experimental observations^28,31^.

### Simulation results

#### Single-subject MEP amplitude

To validate Eq. (5), we simulated 10,000 single subjects with non-normally distributed MEPs at four levels of ${CV}_{trials}$ (0.25, 0.50, 0.75 and 1.00). For each subject, we simulated 100 trials drawn from an independent lognormal distribution with mean ${\mu }_{trials}$ = 1.0 mV and standard deviation ${\sigma }_{trials}$ = 0.25, 0.5, 0.75 or 1.00 mV, with a corresponding skewness = 0.77, 1.63, 2.67, 4.0. The lognormal distribution was obtained as the exponential of a normal distribution with mean

14 $$\mu =\text{log}\left(\frac{{\mu }_{trials}^{2}}{\sqrt{{\mu }_{trials}^{2}+{\sigma }_{trials}^{2}}}\right),$$

and variance

15 $${\sigma }^{2}=\text{log}\left(1+\frac{{\sigma }_{trials}^{2}}{{\mu }_{trials}^{2}}\right).$$

**Figure 1:**
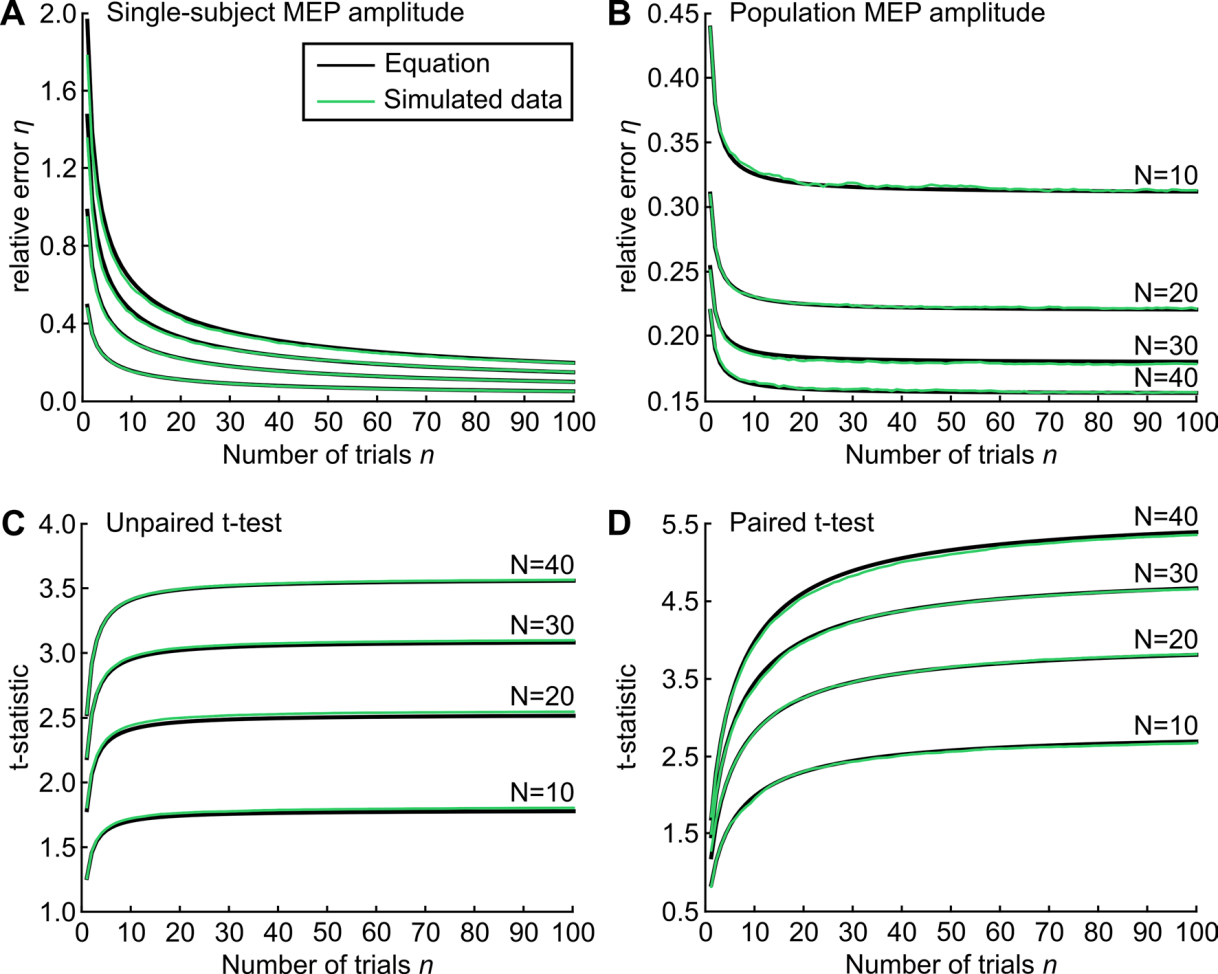
Number of trials for single-subject MEP amplitude, population MEP amplitude and hypothesis testing. (**A**) Single-subject MEP amplitude. With a given number of trials $n$ (x-axis), the single-subject MEP amplitude is expected to be with 95% probability (i.e. ${z}_{1-\alpha /2}$ = 1.96) within a relative error $\eta$ (y-axis) around the true average, depending on the coefficient of variation (${CV}_{trials}$= 0.25, 0.50, 0.75, 1.0). The lines represent Eq. (5) (black) and 10,000 single subjects simulated with lognormally distributed MEP amplitudes (green). (**B**) Population MEP amplitude. Representative example with ${\mu }_{subjects}$ = 1 mV, ${\sigma }_{subjects}$ = 0.5 mV, ${\sigma }_{trials}$ = 0.5 mV. With a given number of trials $n$ (x-axis), the population MEP amplitude is expected to be with 95% probability (i.e. ${z}_{1-\alpha /2}$ = 1.96) within a relative error $\eta$ (y-axis) around the true average, depending on the number of subjects ($N$ = 10, 20, 30, 40). The lines represent Eq. (9) (black) and 10,000 populations of subjects simulated with lognormally distributed MEP amplitudes (green). (**C**) Unpaired t-test. Representative example with ${\mu }_{subjects1}$ = 1.4 mV, ${\mu }_{subjects2}$ = 1.0 mV, ${\sigma }_{subjects}$ = 0.5 mV, ${\sigma }_{trials}$ = 0.5 mV and $r$ = 0. The *t* statistic is plotted as a function of the number of trials $n$, depending on the number of subjects ($N$= 10, 20, 30, 40). The lines represent Eq. (10) (black) and 10,000 populations of subjects simulated with lognormally distributed MEP amplitudes (green). (**D**) Paired t-test. Representative example with ${\mu }_{subjects1}$ = 1.2 mV, ${\mu }_{subjects2}$ = 1.0 mV, ${\sigma }_{subjects}$ = 0.5 mV, ${\sigma }_{trials}$ = 0.5 mV and $r$ = 0.9. The *t* statistic is plotted as a function of the number of trials $n$, depending on the number of subjects ($N$ = 10, 20, 30, 40). Lines as in (**C**). Note that equations (black lines) and simulated data (green lines) are highly overlapping.

We then calculated the cumulative average MEP amplitude for each subject. We finally calculated the 95th percentile of the distribution across subjects of the absolute errors of the cumulative average estimating the true average (divided by 1 mV), as a function of the number of trials $n$. This 95th percentile was used as an estimate of the relative error $\eta$ of the single-subject MEP amplitude. Note that this means that we considered a 95% probability of inclusion of the cumulative average within the relative error $\eta$ from the true average [i.e. ${z}_{1-\alpha /2}$ = 1.96 in Eq. (5)]. The comparison between the simulated data and Eq. (5) is provided in Fig. 1A.

#### Population MEP amplitude

To validate Eq. (9), we simulated 10,000 populations of $N$ = 10, 20, 30 and 40 subjects. For each population of subjects, the single-subject MEP amplitude ${\mu }_{trials}$(*s*) of each subject $s$ was drawn from a lognormal distribution with mean ${\mu }_{subjects}$ = 1.0 mV (i.e. the true population MEP amplitude) and standard deviation ${\sigma }_{subjects}$ = 0.5 mV (skewness = 1.63). For each subject $s$ within each population, we simulated 100 trials drawn from an independent lognormal distribution with mean ${\mu }_{trials}$(*s*) and standard deviation ${\sigma }_{trials}$ = 0.5 mV. We then calculated the cumulative population MEP amplitude for each population of subjects. Finally, we calculated the 95th percentile of the distribution across subjects of the absolute errors of the cumulative population MEP amplitude, estimating the true population MEP amplitude (divided by 1 mV), as a function o the number of trials $n$. This 95th percentile (i.e. ${z}_{1-\alpha /2}$ = 1.96 in Eq. (9)) was used as an estimate of the relative error $\eta$ of the population MEP amplitude. The comparison between the simulated data and Eq. (9) is provided in Fig. 1B.

#### T-statistic for hypothesis testing

To validate Eq. (10), we simulated 10,000 population pairs of $N$ = 10, 20, 30 and 40 subjects each. For each population pair, the single-subject MEP amplitude ${\mu }_{trials}\left({s}_{i}\right)$ of each subject ${s}_{i}$ (with $i$ = 1 or 2) was drawn from a bivariate lognormal distribution with either

(i) means ${\mu }_{subjects1}$ = 1.4 mV and ${\mu }_{subjects2}$ = 1.0 mV, standard deviation ${\sigma }_{subjects}$ = 0.5 mV and covariance 0 (unpaired t-test), or
(ii) means ${\mu }_{subjects1}$ = 1.2 mV and ${\mu }_{subjects2}$ = 1.0 mV, standard deviation ${\sigma }_{subjects}$ = 0.5 mV and covariance $r$* 0.25, with $r$= 0.9 (paired t-test).

The bivariate lognormal distribution was obtained as the exponential of a bivariate normal distribution with means

16 $${\mu }_{1}=\text{log}\left(\frac{{\mu }_{subjects1}^{2}}{\sqrt{{\mu }_{subjects1}^{2}+{\sigma }_{subjects}^{2}}}\right),$$

17 $${\mu }_{2}=\text{log}\left(\frac{{\mu }_{subjects2}^{2}}{\sqrt{{\mu }_{subjects2}^{2}+{\sigma }_{subjects}^{2}}}\right),$$

variances

18 $${\sigma }_{1}^{2}=\text{log}\left(1+\frac{{\sigma }_{subjects}^{2}}{{\mu }_{subjects1}^{2}}\right),$$

19 $${\sigma }_{2}^{2}=\text{log}\left(1+\frac{{\sigma }_{subjects}^{2}}{{\mu }_{subjects2}^{2}}\right)$$

and covariance

20 $$\rho {\sigma }_{1}{\sigma }_{2}={\text{log}}\left(1+\frac{r{\sigma }_{subjects}^{2}}{{\mu }_{subjects1}{\mu }_{subjects2}}\right).$$

Note that we considered the unpaired t-test with equal sample sizes as a special case of the paired t-test (with null covariance). For each subject ${s}_{i}$ within each population pair, we simulated 100 trials drawn from a lognormal distribution with mean ${\mu }_{trials}$(*s*_*i*_) and standard deviation ${\sigma }_{trials}$ = 0.5 mV, and we calculated the cumulative average MEP amplitude across trials. For each population pair, we then computed the average and standard deviation across subjects of the cumulative MEP amplitude differences. To reduce bias, the estimate of the standard deviation was divided by the following correction factor:

21 $${\text{c}}_{4}\left(N\right)=1-\frac{1}{4N}-\frac{7}{32{N}^{2}}-\frac{19}{128{N}^{3}}.$$

Population averages and standard deviations of the cumulative MEP amplitude differences were then averaged across population pairs. The *t* statistic was estimated with the standard formula, as a function of the number of simulated trials $n$ and simulated subjects $N$:

22 $$t\left(n,N\right)=\frac{\stackrel{-}{d}(n,N)}{{s}_{d}(n,N)/\sqrt{N}},$$

where $\stackrel{-}{d}(n,N)$ and ${s}_{d}$ represent the mean and standard deviation of the cumulative MEP amplitude differences averaged across all population pairs. The comparisons between the simulated data and Eq. (10) are provided in Fig. 1C (unpaired) and in Fig. 1D (paired).

### Experimental results

#### Experiment 1

**Figure 2:**
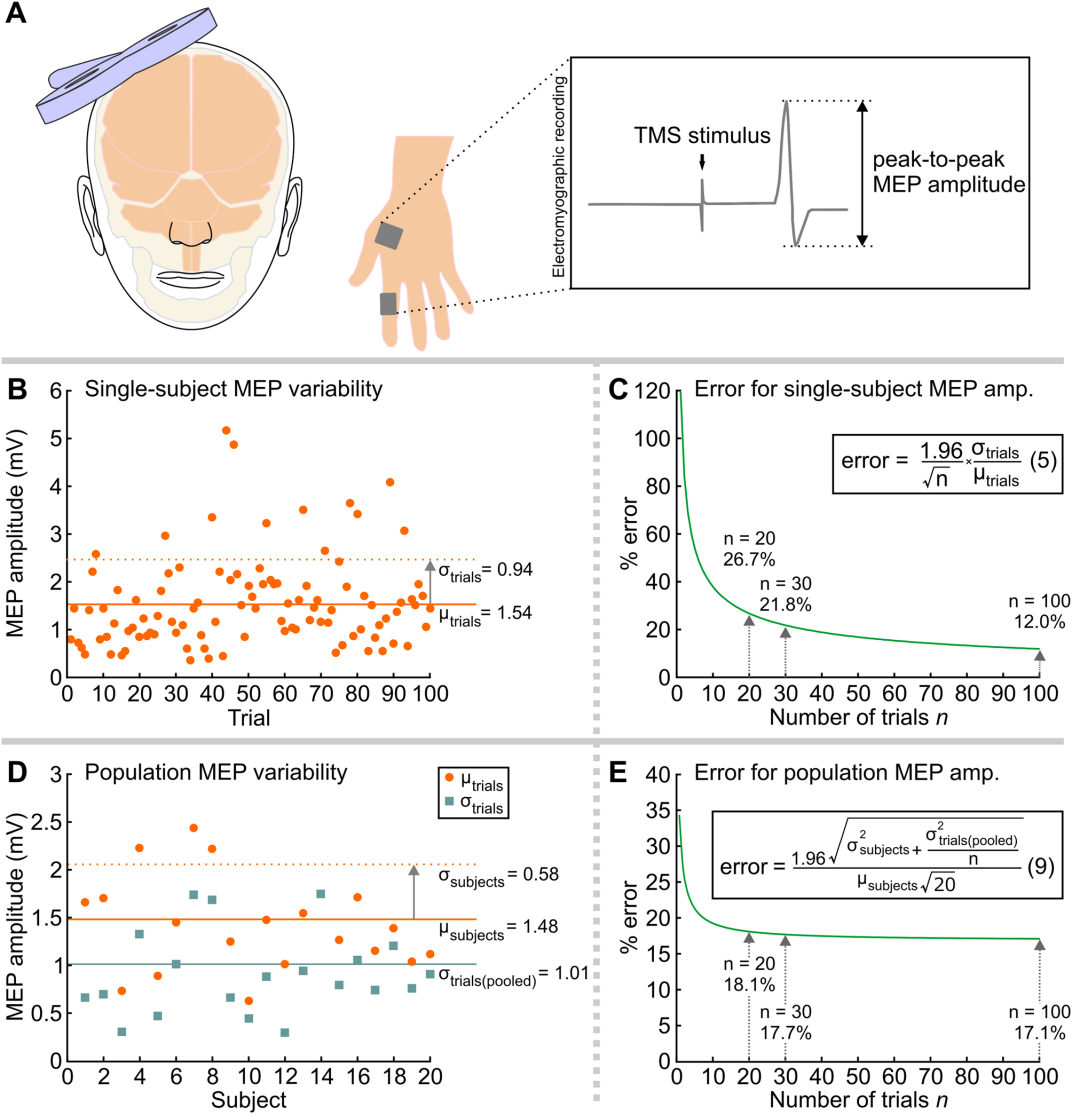
Validation with experimental data from Experiment 1. (**A**) Schematic experimental set up using TMS on the primary motor cortex inducing MEPs in the contralateral FDI muscle. A representative example of a recorded MEP is shown. (**B**) Peak-to-peak MEP amplitudes (mV) from one representative subject showing all 100 trials. (**C**) Experimental application of Eq. (5). (**D**) Average peak-to-peak MEP amplitude (${\mu }_{trials}$) and average standard deviation (${\sigma }_{trials}$) from all subjects ($N$= 20) are represented. (**E**) Experimental application of Eq. (9). (**C,E**) For a 95% c.i., ${\alpha }$= 0.05 and ${z}_{1-{\alpha }/2}$= 1.96.

In the first experiment we addressed the relatively simple problem of estimating MEP amplitude (Fig. 2A). In 20 subjects we set a stimulus intensity intended to evoke approximately 1–1.5 mV MEPs and we delivered 100 single pulses of TMS to the cortical location ('hot spot') representing the FDI. Note that 100 trials is an arbitrary number that is considerably higher than that which is typically used in TMS protocols. Importantly, we did not control for possible attentional drifts over the approximate 10 min required to complete the 100-trial protocol, but we are assuming stationarity for simplicity.

Were 100 trials sufficient to estimate single-subject MEP amplitude? The estimated MEP variability within subjects (${\widehat{\text{CV}}}_{trials}$) was 0.61 (range 0.29 to 0.87). According to Eq. (3), if we wanted to guarantee that the estimated single-subject MEP amplitude was with 95% probability (i.e. ${z}_{1-{\alpha }/2}$ = 1.96) within an arbitrary error of ± 10.0% (i.e. $\eta$ = 0.1) from the true MEP amplitude, we should have increased the number of trials to 143 (range 33–291). Yet Eq. (5) indicates that with our 100 trials the actual difference from the true MEP amplitude was not much higher, just ± 12.0% (range 5.7–17.1%). Using only 30 or 20 trials, the error would increase to ± 21.8% and ± 26.7%, respectively (Fig. 2B,C).

Were 100 trials sufficient to estimate population MEP amplitude? The estimated MEP variability between subjects with 100 trials ${\widehat{CV}}_{subjects}$ was 0.39. Accordingly, Eq. (9) indicates that the estimated population MEP amplitude was with 95% probability within an error of ± 17.1% from the true population MEP amplitude. Importantly, this error would not increase much if the number of trials was decreased to 30 (± 17.7%), 20 (± 18.2%), 10 (± 19.3%.) or even 5 (± 21.4%), (Fig. 2D,E), and it virtually would not decrease further if we had an infinite number of trials (± 16.9%).

#### Experiment 2

As a representative example of hypothesis testing, we considered the problem of designing an experiment to test whether stimulus intensity affects MEP amplitude (although we actually know that it does). We thus decide to deliver stimuli at two intensities commonly used in stimulus–response curves: 110% and 120% of the RMT^9,16,26^, and we use the results of Experiment 1 to make predictions for the following question: how many trials and subjects do we need to detect a difference in MEP amplitude between 110%RMT and 120%RMT?

In Experiment 1, the actual stimulus intensity employed was 122.5 ± 11.8% of the RMT, which elicited a population MEP amplitude ${\widehat{\mu }}_{subjects}$ = 1.48 mV, with an estimation error of 17.1%, a pooled within-subjects MEP variability ${\widehat{\sigma }}_{trials\left(pooled\right)}$ = 1.01 mV and an estimated asymptotic between-subjects MEP variability ${\widehat{\sigma }}_{subjects}$ = 0.57. We thus make the following conservative estimations. (a) With 120%RMT intensity we will obtain a population MEP amplitude ${\mu }_{subjects1}$ = 1.48*(1 $-$ 0.171) = 1.23 mV (i.e. the lower confidence limit from experiment 1). (b) With 110%RMT we will obtain a population MEP amplitude ${\mu }_{subjects2}$ = 1.23/2 = 0.62 mV. (c) Both within-subject and between-subjects MEP variability will be the same at 110%RMT and at 120%RMT, i.e. ${\sigma }_{trials\left(pooled\right)}$ = 1.01 mV and ${\sigma }_{subjects}$ = 0.57 mV. (d) The asymptotic correlation between MEPs obtained at 110%RMT and at 120%RMT will be $r$ = 0.61. The latter was estimated from the split-half correlation of the first 40 trials in Experiment 1 (i.e. the correlation of the mean MEPs estimated from the first 20 trials with the mean MEPs estimated from the next 20 trials), eliminating one outlier.

**Figure 3:**
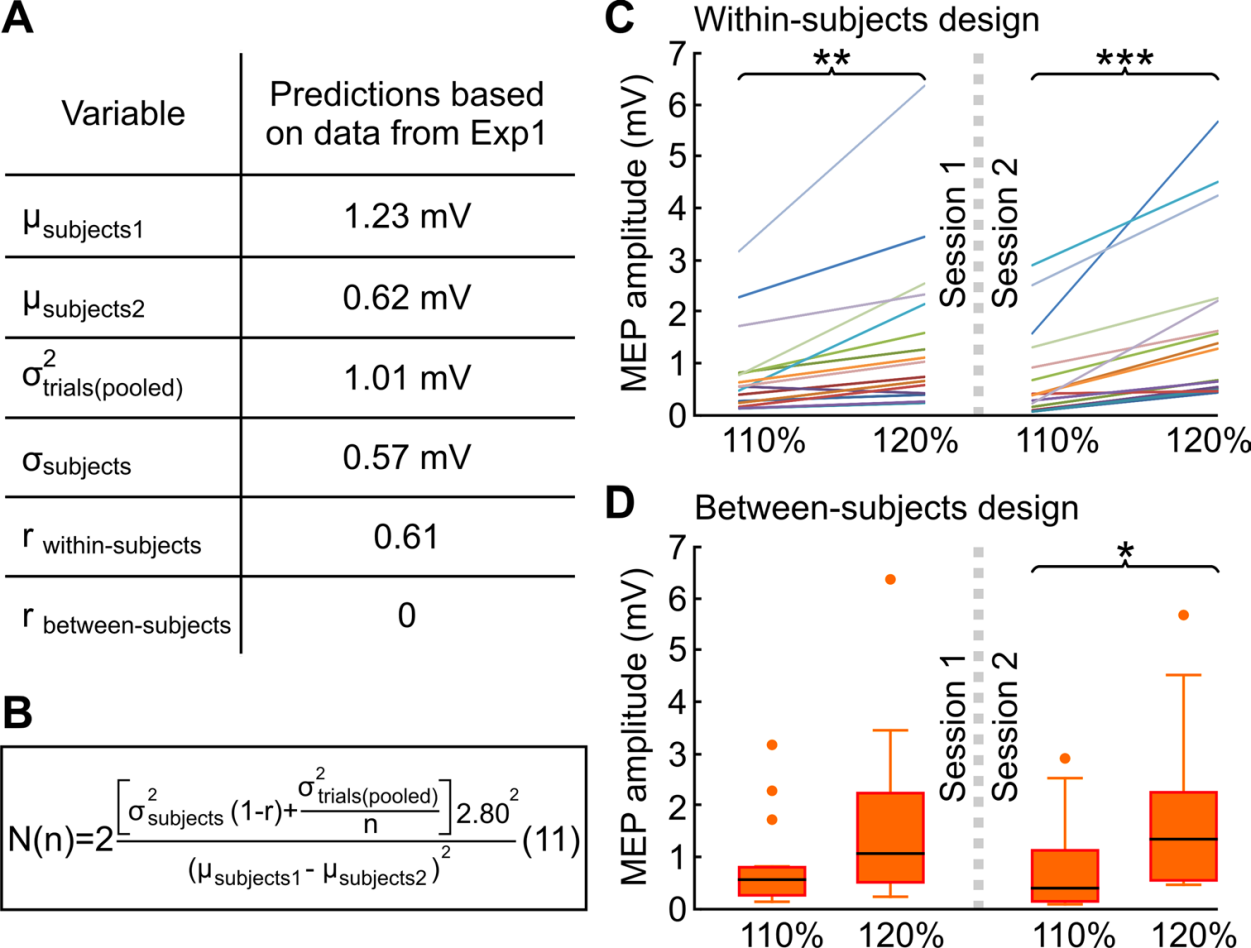
Experimental validation of Eq. (11). (**A**) Estimations based on data from Experiment 1 to calculate the number of trials needed to detect a difference in MEP amplitude between 110%RMT and 120%RMT. (**B**) With the numbers from (**A**), Eq. (11) determines that to detect a significant difference using 10 (or 30) trials in a within-subjects design it would require 10 (or 7) subjects, whereas in a between-subjects design it would require 18 (or 16) subjects. $\alpha$ = 0.05, $\beta$ = 0.20 (i.e. ${z}_{\text{1} - \alpha \text{/2}}+ {z}_{{1}-\beta }$ = 2.80). (**C**) Experimental validation of predictions made by Eq. (11) on MEP amplitude (mV) measured at two different intensities (110%RMT and 120%RMT) for a within-subjects design with 10 trials per TMS intensity and 16 subjects. The session was repeated twice (Experiment 2). Each colored line represents a single subject. Paired t-test; **p < 0.01; ***p < 0.001. (**D**) Same as in (**C**) assuming a between-subjects design (i.e. two groups of 16 subjects), showing expected lower statistical power. Results are shown as box plots (horizontal lines: median (Q2), first quartile (Q1) and third quartile (Q3); whiskers: minimum and maximum value excluding outliers; outliers: points larger than Q3 + 1.5(Q3–Q1) or smaller than Q1–1.5(Q3–Q1). Unpaired t-test; *p < 0.05.

With the above numbers (Fig. 3A), Eq. (11) indicates that in order to detect a significant difference in MEP amplitude between 110%RMT and 120%RMT, with type-I error ${\alpha }$ < 0.05 and type-II error $\beta$ < 0.20 (i.e. power > 0.80), with infinite trials we would need only 6 subjects in a within-subjects design. This minimum number of subjects would increase to 7, 8, 10, and 14 with 30, 20, 10, and 5 trials, respectively. If instead we planned to perform the experiment in a between-subjects design ($r$ = 0, i.e. one group tested at 110%RMT and the other group tested at 120%RMT), Eq. (11) tells us that with infinite trials we would need at least 14 subjects per group, which would increase to 16 and 18 subjects with 30 or 10 trials, respectively (Fig. 3B).

We decided to perform Experiment 2 in a within-subjects design with 10 trials per intensity and 16 subjects, in order to have more than enough power to detect a significant difference in a within-subject design (even with half of the trials), and almost sufficient power if assuming a between-subjects design. The two stimulus intensities (i.e. 110%RMT and 120%RMT) were delivered in the same experimental session, and the experiment was repeated twice to verify the consistency of the statistical results. As expected, MEP amplitude was greater at 120%RMT compared to 110%RMT both in the first session (1.57 ± 1.59 mV vs. 0.81 ± 0.85 mV) and in the second session (1.79 ± 1.64 mV vs. 0.76 ± 0.89 mV). Considering only the first 10 subjects (i.e. the minimum number of subjects to detect a significant difference as suggested by Eq. (11)), MEP amplitude was significantly higher with 120%RMT compared to 110%RMT, both in the first experimental session (paired t-test, $p$= 0.010) and in the second one ($p$ = 0.044). The $p$-values decreased as expected considering the entire sample of 16 patients, both in the first experimental session ($p$ = 0.003) and in the second one ($p$ < 0.001) (Fig. 3C). As predicted, the difference remained significant even when only 5 trials were used, both in the first session ($p$ = 0.007) and in the second one ($p$ = 0.001). Conversely, if we assumed that the experiment was performed in a between-subjects design (i.e. two groups of 16 subjects), the $p$-values reached significance in the second session (unpaired t-test, $p$ = 0.034), but not in the first one ($p$ = 0.10), consistent with the lower statistical power that had been expected (Fig. 3D).

## Discussion

We presented a general framework of simple equations that show how the number of trials affects single-subject MEP amplitude, population MEP amplitude, hypothesis testing and test–retest reliability in TMS experiments. The equations were derived analytically, validated with Monte Carlo simulations, and applied to two sets of experimental data in a representative manner.

### Analytical results

A number of recent experimental studies suggested that the minimum number of trials for estimating MEP amplitude would be around 30 trials^26,28,29^. However, we analytically showed that with the empirical approach used in these studies the estimated minimum number of trials essentially depends on total number of trials available ${n}_{max}$[Eqs. (1) and (2)] and does not depend on the trial-to-trial variability [Eq. (1)]. This probably explains why in these studies the estimated minimum number of trials ${n}_{opt\_ci}$ for MEPs collected at 120%RMT was higher when ${n}_{max}$ was 40 (${n}_{opt\_ci}$ = 29–31)^26,28,29^, compared to when ${n}_{max}$ was 30 (${n}_{opt\_ci}$ = 21)^27^. Previous experimental estimates of the minimum number of trials to reliably estimate single-subject MEP amplitude thus do not lend themselves to generalization.

Equation (3) formalizes the intuition that the minimum number of trials to estimate single-subject MEP amplitude should depend on the trial-to-trial variability in the specific experimental conditions and on the acceptable statistical error defined by the experimenter^14^. Indeed, depending on stimulus intensity and on the stimulus–response curve of the individual subject in the specific experimental condition^18,38^, MEP amplitude has a different trial-to-trial variability, as measured by the coefficient of variation (${CV}_{trials}$)^9,16,39,40^. This affects the minimum number of trials required to estimate single-subject MEP amplitude, which is proportional to the square of ${CV}_{trials}$. When the same equation is resolved in terms of the acceptable statistical error [Eq. (5)], it becomes explicit that increasing the number of trials dramatically reduces the error when only a few trials are available, but it offers a progressively smaller advantage as the number of trials increases (Fig. 1A). Nevertheless, the present study warns us that, if the acceptable error is low, in many experimental conditions estimating single-subject MEP amplitude may require substantially more trials than previously suggested (but maintaining stationary conditions may become a challenge). However, increasing the number of trials can only improve the test–retest reliability of MEP amplitude up to a limit [Eq. (13)], in agreement with previous experimental results^28,31^. This is important, for example, for possible diagnostic applications^41,42^, or for assessing the reproducibility of non-invasive brain stimulation techniques in individual subjects^43–45^.

Equations (9), (10) and (11) define the impact of the number of trials for estimating population MEP amplitude and for hypothesis testing. Importantly, the non-linearity of the stimulus–response curve and its between-subjects variability contribute to both the between-subjects MEP amplitude variability ${\upsigma }_{subjects}$ and the pooled within-subjects MEP amplitude variability ${\upsigma }_{trials}$. This has a much higher impact on the minimum number of subjects than on the minimum number of trials required to estimate population MEP amplitude within a certain error or to detect a significant difference in hypothesis testing. In fact, the number of trials and trial-to-trial variability within subjects have a relatively minor impact on the estimation of population MEP amplitude, which mostly depends on the variability between subjects and on the number of subjects [Eq. (9); Fig. 1B]. Similarly, hypothesis testing is markedly more dependent on the number of subjects than on the number of trials [Eqs. (10) and (11)], particularly in unpaired experimental designs ($r$= 0; Fig. 1C). In paired designs (0 < $r$ < 1), importantly, the number of trials becomes progressively more relevant if the asymptotic correlation $r$ between repeated measures is higher (Fig. 1D). Nevertheless, even for highly reliable paired conditions (e.g. $r$ = 0.9), a decrease in number of trials can always be compensated by an increase in number of subjects. In general, unless very few trials are used, increasing the number of trials will only induce a minor improvement in statistical power and reproducibility of comparisons between subjects (e.g. patients vs. controls) or within subjects (e.g. effect of an intervention). If more statistical power is needed, then the number of subjects rather than trials should be increased. Indeed, if sufficient subjects are available, theoretically the minimum number of trials per subject to detect any difference is always $n$ = 1.

### Simulation results

MEP amplitudes are typically not normally distributed. However, our analytical framework does not assume that MEP amplitudes are normally distributed: it assumes that the sample estimates of MEP amplitude means are normally distributed. Normal distribution of sample means is indeed guaranteed when the samples are normally distributed, but it also guaranteed by the central limit theory even when the samples are not normally distributed, if sufficient trials are available. To support this point, we validated Eqs. (5), (9) and (10) with Monte Carlo simulations that assumed lognormal distribution of single-trial MEP amplitudes within subjects and of single-subject MEP amplitude across subjects (Fig. 1). The results obtained with lognormal simulated data are highly consistent with the analytical equations. Note that very minor deviations from Eq. (5) are observed in the lognormal simulations, as expected, only with few trials and heavily skewed simulated data (skewness = 4 in Fig. 1; as a reference, the average skewness in Exp. 1 was 1.20, range [0.48–2.25]). Therefore, with low numbers of trials and/or in the presence of “outliers”, the estimates obtained with the equations may be more accurate after normalizing the data, e.g. via an appropriate Box-Cox transformation^46,47^. Still, in most cases the equations can be readily applied to raw MEP data.

### Experimental results

We provided a step-by-step application of the equations to estimate single-subject MEP amplitude and population MEP amplitude in a dataset of 100 MEP trials recorded in 20 subjects (Experiment 1). Our results show that 100 trials were sufficient to keep the estimation error of MEP amplitude below ± 20% in all our subjects, and they suggest that most experimental paradigms employing 20–30 trials (including ours) implicitly accept relatively large estimation errors for single-subject MEP amplitude. On the other hand, 100 trials were not only sufficient, but also unnecessarily high to estimate population MEP amplitude. The experimental results confirm that in the estimation of single-subject MEP amplitude the concept of “minimum number of trials” essentially depends on the error that is considered acceptable by the experimenter and the variability of MEPs in the individual subject. Conversely, the number of trials plays little role in the estimation of population MEP amplitude, which is more dependent on number of subjects.

We then used the data from Experiment 1 to define the optimal number of trials and subjects to be used in a representative experiment designed to detect significant MEP amplitude differences between two stimulus intensities (i.e. 110%RMT vs. 120%RMT; Experiment 2). Our results provide a practical example of how Eq. (11) can be used as a tool to assess the impact of the number of trials when designing new experiments. The same reasoning can be used to estimate the impact of the number of trials on experiments aiming to assess differences in MEP amplitude between groups of subjects (e.g. patients vs. controls) or changes in MEP amplitude before and after an intervention (e.g. non-invasive brain stimulation protocols).

Importantly, the equations have broad applicability and are generally valid for all experimental measures and conditions dealing with multiple trials per subject and populations of subjects. Within the TMS field, for example, the same equations can be directly applied to any measure of MEP amplitude (e.g. peak-to-peak, area, modulus, etc.) at any intensity on the stimulus–response curve, and to other single-pulse measures such as the silent period. Different experimental conditions (e.g. at rest, in activation, during a task, etc.) can be readily reflected in the equations by entering the corresponding values of within and between-subjects variability. The framework can also be extended, at least in principle, to more complex measures, such as the steepness of the stimulus–response curve and paired-pulse TMS measures. For these measures, however, some effort may be necessary to properly estimate within and between-subjects variability as a function of the number of trials. Indeed, the same equations and reasoning can also be applied to other fields (e.g. reaction times in behavioral tasks, etc.).

### Practical recommendations

The aim of this study was not to provide rule-of-thumb answers that may be valid only in specific experimental conditions, but to offer a more general framework to inform the decision about how many trials to use under different experimental conditions. Still, we can provide the following practical recommendations:

1. For estimating single-subject MEP amplitude, the minimum number of trials largely depends on the variability of the subject in the exact experimental conditions, and on the error considered acceptable by the experimenter. Equation (3) can be used to directly estimate the minimum number of trials, given the variability and the acceptable error. Equation (5) can be used to estimate the error, given the variability and the number of trials. An important caveat is that the estimate of single-subject MEP amplitude and its corresponding error refer only to the moment of the test. Their ability to represent the subject in general depends on test–retest reliability [Eq. (13)]. With this in mind, the general recommendation for estimating single-subject MEP amplitude is to use a relatively high number of trials.
2. For hypothesis testing, the number of trials plays a relatively minor role. Equation (11) can be used to explicitly estimate the impact of the number of trials in a power analysis. The general recommendation for hypothesis testing is to use at least few trials and to include a relatively high number of subjects.

Overall, we hope these simple equations will offer a useful tool to solve the issue of maximizing the number of trials and minimizing experimental time in many experimental situations, and to clarify the impact played by the number of trials on the design and reproducibility of past and future experiments.

## Methods

### Subjects

The study was performed according to the declaration of Helsinki and approved by the local Ethics Committee (Comité Ético de Investigación de HM Hospitales). We recruited 27 right-handed healthy participants (15 females; mean age ± standard deviation: 27.3 ± 5.7 years, 20–40 years old, 85% non-smokers) with a negative history of neurological or psychiatric conditions and medication-free at the time of the study. All subjects gave their informed consent.

### Electromyographic recordings

We recorded EMG activity from the first dorsal interosseous (FDI) using disposable surface electrodes. EMG signals were band-pass filtered (2 Hz–2 kHz) and amplified (× 1000; D360, Digitimer Ltd, UK) and single trials were digitized (sample rate 5 kHz) using a CED 1401 A/D converter and Signal 5 software (Cambridge Electronic Design, Cambridge, UK). EMG signals were monitored online via visual feedback on a computer screen.

### Transcranial magnetic stimulation

We used a 70-mm figure-eight-shaped magnetic coil connected to a Magstim 200^2^ stimulator (Magstim Co. Ltd, UK) to perform monophasic single-pulse TMS. The coil was held tangential to the scalp with the handle oriented backwards and 45° from the midline. The induced current presented a posterior-anterior (PA) direction activating preferentially I1 waves^48,49^. Both experiments were performed using a frameless neuronavigation system (BrainSight, Rogue Research, Canada) to guide the coil position with the help of a magnetic resonance imaging template in standard space. For all experiments we measured the individual RMT, defined as the minimum TMS output intensity required to evoke a MEP peak-to-peak amplitude of ≥ 0.05 mV in five out of 10 consecutive trials in the resting FDI. We delivered TMS single pulses with 6 s ± 10% as inter-trial interval. This inter-trial interval was chosen to minimize the carryover effects in the initial transient state observed at intervals ≤ 5 s^24,50^ and to be consistent with our recent studies^51–53^.

### Experimental procedures

We performed two independent experiments. Eighteen subjects participated in one experiment and 9 subjects participated in both. Subjects sat in a comfortable chair and were instructed to relax both arms and hands on a pillow keeping their eyes open for the duration of the experiment. Experiment 1 ($n$ = 20; 11 females; mean age 27.7 ± 5.6 years): For each subject we determined the FDI 'hot spot' in the right motor cortex and measured the RMT. After establishing the TMS output intensity that evoked a peak-to-peak MEP amplitude of 1–1.5 mV, we recorded 100 MEPs at rest at that intensity. Experiment 2 ($n$ = 16; 8 females; mean age 25.9 ± 4.8 years): Each subject performed two identical sessions, 7 days apart. In each session we determined the individual FDI 'hot spot' in the right motor cortex. We measured the RMT and recorded 40 MEPs at rest at different TMS output intensities (110%, 120%, 130%, and 140%RMT; randomized). Only the data from 110%RMT and 120%RMT were used in this study. In both experiments, single-trial MEP amplitude was estimated as peak-to-peak amplitude of recorded the EMG signal.

### Derivation of Eq. (1)

The optimal number of trials ${n}_{op{t}_{ci}}$ estimated by the inclusion of the cumulative average ${\widehat{\mu }}_{trials}\left(n\right)$ within a 95% confidence interval around the sample average ${\widehat{\mu }}_{trials}\left({n}_{max}\right)$, as used empirically in previous experimental studies^26–29,31^, can be defined analytically. We will refer to the sample average ${\widehat{\mu }}_{trials}\left({n}_{max}\right)$ simply as ${\widehat{\mu }}_{trials}$, and to the true average as ${\mu }_{trials}$.

First, the half width of the 95% confidence interval around the sample average ${\widehat{\mu }}_{trials}$ is simply ${z}_{1-{{\alpha }}_{ci}/2}SE\left({n}_{max}\right)$, where ${z}_{1-{{\alpha }}_{ci}/2}$ is the critical value (for a 95% c.i., ${{\alpha }}_{ci}$ = 0.05 and ${z}_{1-{{\alpha }}_{ci}/2}$ = 1.96) and $SE\left({n}_{max}\right)$ is the standard error of the estimate of the true average ${\mu }_{trials}$ with the maximum number of trials available ${n}_{max}$. Second, we can define the ‘inclusion of the cumulative average’ within the above confidence interval around the sample average in probabilistic terms, as the confidence interval of the estimate of the sample average made by cumulative average: ${z}_{1-{\alpha }/2}{SE}_{sample}\left(n\right)$, where ${z}_{1-{\alpha }/2}$ is the critical value defined by the probability of inclusion ${p}_{incl}$ (i.e. ${\alpha }=1-{p}_{incl}$) and ${SE}_{sample}\left(n\right)$ is the standard error of the cumulative average estimating the sample average with *n* samples. Note that the above cited studies empirically used ${p}_{incl}$ = 1, which would correspond to a theoretical ${\text{z}}_{1-{\alpha }/2}$ =  + ∞, but in practice corresponded to an arbitrary ${p}_{incl}$ < 1 that depends on the number of subjects.

The optimal number of trials ${n}_{opt\_ci}$ is then defined as the number of trials at which the confidence interval of the estimate of the sample average made by the cumulative average equals the confidence interval of the estimate of the true average made by the sample average, i.e.

23 $${{n}_{opt\_ci}=n : z}_{1-{\alpha }/2}{SE}_{sample}\left(n\right)={z}_{1-{\alpha }_{ci}/2}SE\left({n}_{max}\right).$$

In Eq. (23), $SE\left({n}_{max}\right)$ is given by the well-known formula:

24 $$ SE\left( {n_{max} } \right) = \frac{{\hat{\sigma }_{trials} }}{{\sqrt {n_{max} } }}, $$

where ${\widehat{\sigma }}_{trials}$ is the standard deviation of MEP amplitude across trials.

${SE}_{sample}\left(n\right)$ is somewhat less straightforward. Let $\varepsilon (n)$ be the error in the estimate of the sample average made by the cumulative average with $n$ < ${n}_{max}$, i.e.

25 $${\widehat{\mu }}_{trials}\left(n\right)={{\widehat{\mu }}_{trials}\left({n}_{max}\right)+\varepsilon \left(n\right)}.$$

From the decomposition of variances, it follows that:

26 $${\text{Var}[\widehat{\mu }}_{trials}\left(n\right)]={\text{Var}\left[{\widehat{\mu }}_{trials}\left({n}_{max}\right)\right]+\text{Var}[\varepsilon \left(n\right)]},$$

where $Var[\varepsilon \left(n\right)]$ is the variance of the cumulative average estimating the sample average. Since the standard deviation of an estimator (in this case the cumulative average as an estimator of the sample average) is by definition the standard error of the estimator, we can write:

27 $$\text{Var}\left[\varepsilon \left(n\right)\right]={SE}_{sample}{\left(n\right)}^{2}.$$

${Var[\widehat{\mu }}_{trials}\left(n\right)]$ is the variance of the cumulative average estimating the true average, i.e.

28 $$\text{Var}\left[{\widehat{\mu }}_{trials}\left(n\right)\right]=SE{\left(n\right)}^{2}=\frac{{{\widehat{\sigma }}_{trials}}^{2}}{n}$$

and ${Var[{\widehat{\mu }}_{trials}\left({n}_{max}\right)]}$ is the variance of the sample average estimating the true average, i.e.

29 $$\text{Var}\left[{\widehat{\mu }}_{trials}\left({n}_{max}\right)\right]=SE{\left({n}_{max}\right)}^{2}=\frac{{{\widehat{\sigma }}_{trials}}^{2}}{{n}_{max}}.$$

The variance of the cumulative average estimating the sample average ${SE}_{sample}{\left(n\right)}^{2}$ can thus be readily obtained by subtracting the variance of the sample average to the variance of the cumulative average estimating the true average, i.e.

30 $$S{E}_{sample}{\left(n\right)}^{2}=SE{\left(n\right)}^{2}-SE{\left({n}_{max}\right)}^{2} =\frac{{{\widehat{\sigma }}_{trials}}^{2}}{n}-\frac{{{\widehat{\sigma }}_{trials}}^{2}}{{n}_{max}}= {{\sigma }_{trials}}^{2}\left(\frac{1}{n}-\frac{1}{{n}_{max}}\right).$$

Substituting (24) and (30) in (23) we obtain:

31 $${{n}_{opt\_ci}=n : z}_{1-{\alpha }/2} {\widehat{\sigma }}_{trials}\sqrt{\frac{1}{n}-\frac{1}{{n}_{max}}} =\frac{{z}_{1-{{\alpha }}_{ci}/2} {\widehat{\sigma }}_{trials}}{\sqrt{{n}_{max}}},$$

which gives

32 $${n}_{opt\_ci}=\frac{{n}_{max}}{1+{\left(\frac{{z}_{1-{{\alpha }}_{ci}/2}}{{z}_{1-{\alpha }/2}}\right)}^{2}},$$

corresponding to Eq. (1).

### Derivation of Eq. (2)

The optimal number of trials ${n}_{opt\_\%diff}$ estimated by the inclusion of the cumulative average within a ± 10% difference around the sample average, as used empirically in one previous study^28^, can also be defined analytically, as follows:

33 $${n}_{opt\_\%diff}=n : {z}_{1-\alpha /2}{SE}_{sample}\left(n\right)=\eta {\widehat{\mu }}_{trials},$$

where ${z}_{1-\alpha /2}{SE}_{sample}\left({n}_{opt\_\%diff}\right)$ is the confidence interval of the estimate of the sample average made by the cumulative average, as in Eq. (23), $\eta$ = 0.1 for ± 10% difference and ${\widehat{\mu }}_{trials}$ is the sample average. Substituting (30) in (33), we obtain:

34 $${n}_{opt\_\%diff}=n : {z}_{1-\alpha /2}{\widehat{\sigma }}_{trials}\sqrt{\frac{1}{n}-\frac{1}{{n}_{max}}}=\eta {\widehat{\mu }}_{trials},$$

which gives:

35 $${n}_{opt\_\%diff}=\frac{1}{\frac{1}{{n}_{max}}+{\left(\frac{{\eta \widehat{\mu }}_{trials}}{{z}_{1-\alpha /2 {\widehat{\sigma }}_{trials}}}\right)}^{2}},$$

corresponding to Eq. (2).

### Derivation of Eq. (10)

To derive Eq. (10), we start from the estimation of the *t* statistic in a paired Student’s t-test, i.e.

36 $$t=\frac{{\widehat{\mu }}_{subjects1}-{\widehat{\mu }}_{subjects2}}{\sqrt{\frac{{\widehat{\sigma }}_{subjects1}^{2}+{\widehat{\sigma }}_{subjects2}^{2}-2\text{cov}\left({\widehat{{\varvec{\mu}}}}_{trials1}, {\widehat{{\varvec{\mu}}}}_{trials2}\right)}{N}}},$$

where ${\widehat{\varvec{\mu} }}_{trials1}$ and ${\widehat{\varvec{\mu} }}_{trials2}$ are vectors of estimated single-subject MEP amplitudes for two repeated measures from the same population of subjects. Note that if we impose $\text{cov}\left({\widehat{\varvec{\mu} }}_{trials1}, {\widehat{\varvec{\mu} }}_{trials2}\right)$ = 0, then Eq. (36) becomes the *t* statistic for an unpaired t-test between two populations with an equal number of subjects.

We assume equal variances (or pool them) so that ${\widehat{\sigma }}_{subjects1}^{2}+{\widehat{\sigma }}_{subjects2}^{2}={2 \widehat{\sigma }}_{subjects}^{2}$, and we model the estimated single-subject MEP amplitudes as:

37 $${\widehat{\varvec{\mu} }}_{trials}={\varvec{\mu} }_{trials}+\varvec{\varepsilon},$$

where ${\varvec{\mu} }_{trials}$ is the vector of true single-subject MEP amplitudes across subjects and $\varvec{\varepsilon}$ is the corresponding error vector for estimating the single-subject MEP amplitude with a limited number of trials. Assuming that the errors are independent, the covariance term can be rewritten as follows:

38 $$cov({\widehat{\varvec{\mu} }}_{trials1},{\widehat{\varvec{\mu} }}_{trials2})=r\left(n\right){\sigma }_{subjects}^{2}\left(n\right)=cov\left({\varvec{\mu} }_{trials1}+{\varvec{\varepsilon} }_{1},{\varvec{\mu} }_{trials2}+{\varvec{\varepsilon} }_{2}\right)=cov\left({\varvec{\mu} }_{trials1},{\varvec{\mu} }_{trials2}\right)=r{\sigma }_{subjects}^{2},$$

where $r\left(n\right)$ and ${\sigma }_{subjects}^{2}\left(n\right)$ are the Pearson’s correlation across repeated measures and the (pooled) variance across subjects with $n$ trials, whereas $r$ and ${\sigma }_{subjects}^{2}$ are the asymptotic Pearson’s correlation across repeated measures and (pooled) variance across subjects with infinite trials. Substituting Eqs. (7) and (38) in Eq. (36), we obtain:

39 $$t(N,n)=\frac{{\widehat{\mu }}_{subjects1}-{\widehat{\mu }}_{subjects2}}{\sqrt{\frac{2\left[{ \sigma }_{subjects}^{2}\left(1-r\right)+\frac{{\sigma }_{trials}^{2}}{n}\right]}{N}}},$$

which corresponds to Eq. (10). Note that

40 $$r=r\left(n\right)\frac{{\sigma }_{subjects}^{2}\left(n\right)}{{\sigma }_{subjects}^{2}}=r\left(n\right)\frac{{\sigma }_{subjects}^{2}\left(n\right)}{{ \sigma }_{subjects}^{2}\left(n\right)-\frac{{\sigma }_{trials}^{2}}{n}}> r\left(n\right).$$

Therefore, $r\left(n\right)$ provides a lower bound for $r$, and $r$ can be estimated from the data. Note that Eq. (40) corresponds to a classic correction for attenuation^54,55^.

## Data Availability

The main code is given within the manuscript in form of equations (which are sufficiently simple to be readily implemented in any spreadsheet or programming language). The experimental data are available upon reasonable request.
